# Soil Legacy Effects of *Chromolaena odorata* and Biochar Remediation Depend on Invasion Intensity

**DOI:** 10.3390/plants14030401

**Published:** 2025-01-29

**Authors:** Jiajun Li, Yulong Zheng, Shukui Chang, Yangping Li, Yi Wang, Xue Chang, Weitao Li

**Affiliations:** 1 Xishuangbanna Tropical Botanical Garden, Chinese Academy of Sciences, Mengla 666303, China; lijiajun@xtbg.ac.cn (J.L.); zhengyl@xtbg.org.cn (Y.Z.); liyp@xtbg.org.cn (Y.L.); s2177587@siswa.um.edu.my (X.C.); 2 School of Ecology and Environmental Sciences, Yunnan University, Kunming 650500, China; yiwang@ynu.edu.cn; 3 University of Chinese Academy of Sciences, Beijing 100049, China; 4 College of Agriculture and Forestry, Puer University, Puer 665000, China; yndl15368307813@163.com; 5 Yunnan Key Laboratory for the Conservation on Tropical Rainforests and Asian Elephants, Xishuangbanna Tropical Botanical Garden, Chinese Academy of Sciences, Mengla 666303, China; 6 Institute of Biological Sciences, University of Malaya, Kuala Lumpur 50603, Malaysia

**Keywords:** biological invasion, invasive plant, soil legacy effect, biochar, soil remediation

## Abstract

The increasing threat of biological invasion poses significant challenges to global ecosystems, necessitating urgent management measures. This study investigated the potential of biochar derived from invasive plant *Chromolaena odorata*, produced through anaerobic digestion, as a tool for mitigating the soil legacy effects of this species and restoring the plant community. Soil samples were collected from artificially constructed plots of invasive plant communities and were subjected to treatments with different levels of fungicide and biochar addition. Potted plant communities replicating the original species composition were established, and biomass were used to evaluate the effectiveness of soil restoration. Our results demonstrated that soil sterilization enhanced plant biomass, with invasive plants showing a more pronounced increase compared to native species, indicating different responses to the soil biota. The addition of biochar influenced plant biomass, with an optimal biochar concentration of 2% of the soil mass, promoting the growth of native plants. The application of biochar in conjunction with soil sterilization facilitated the restoration of native communities in areas with low invasion intensity. Overall, these findings provide valuable insights into the potential of biochar-based strategies for managing invasive plants and restoring ecosystems, underscoring the necessity for further research to optimize field applications and evaluate ecological impacts.

## 1. Introduction

With globalization and increasing human cross-regional communication, more species are being intentionally or unintentionally introduced into new areas [1], where they establish, spread, and ultimately cause serious harm to local ecosystems [2,3]. Invasive plants can profoundly impact local ecosystems by threatening the native biodiversity and affecting local arthropod communities [4,5]. They also pose significant threats to human health, society, and the economy, leading to reduced crop yields, jeopardizing food security, and acting as allergens that endanger human well-being [6]. Furthermore, biological invasion continues worldwide, with the number of invasive species increasing annually [7,8]. Consequently, controlling and managing biological invasion has become an urgent concern in ecological research.

Among the disasters caused by biological invasion, one of the more covert processes occurring underground is the rapid depletion of soil nutrients by invasive species to achieve rapid growth [9]. Invasive species alter the soil microbial community by recruiting large numbers of pathogenic microorganisms [10], profoundly and permanently changing the abiotic and biotic properties of the soil and the function of plant communities [11,12]. Additionally, invasive plants can alter the soil seed bank [13], and ecosystem nitrogen dynamics, including soil nitrogen pools and nitrogen cycling [14,15], have long-term effects on nitrification [16]. Even after the removal of alien plants, the residual soil environment can continue to influence the growth of subsequent plants through what are known as soil legacy effects [17]. These effects determine the subsequent success of alien plant invasion and the reestablishment of native plants, thereby influencing the dynamics of the invaded community [18].

To remove invasive plants from communities, large amounts of herbicides are often used. Among nonchemical methods, the burning of alien plants is common for preventing further spread through reproduction [19]. Invasive plants, despite causing significant ecological damage, sequester substantial amounts of carbon in their tissues, thereby acting as a carbon sink. This capability is attributed to their photosynthetic efficiency [20], relative seedling growth rate [21], photosynthetic nitrogen use efficiency [22], and water use efficiency being greater than those of native plants, enabling invasive plants to accumulate large amounts of biomass within a short period. However, the burning of these plants releases unnecessary carbon dioxide into the atmosphere, exacerbating the greenhouse effect.

Considering these points, developing an environmentally friendly approach that addresses both the removal of alien invasive species and the restoration of local ecosystems after invasion is necessary. Biochar might provide a solution to these issues. Biochar is produced by pyrolyzing biomass such as wood, litter, manure, and plant tissues under oxygen-limited or anaerobic conditions at relatively low temperatures (<700 °C). It possesses high porosity and a large specific surface area, conferring strong adsorption properties [23,24]. Biochar can adsorb allelopathic chemicals [25,26,27], improve soil physicochemical properties and microbial activity, and enhance soil fertility [28,29,30,31,32], thereby potentially mitigating the soil legacy effects of plant invasions.

Processing alien plants into biochar can deactivate the propagules of alien species [33], thus safely and effectively managing the removed plants, securely sequestering them back into the soil carbon pool, and serving as a potential method to mitigate global warming [34]. Additionally, biochar may help rebuild the local plant community by enhancing soil fertility. Combining biochar with fungicides can also eradicate pathogenic microorganisms recruited by alien invasive plants in the soil. The combined use of biochar and fungicides may serve as a method to alleviate soil legacy effects after invasion.

**Figure 1:**
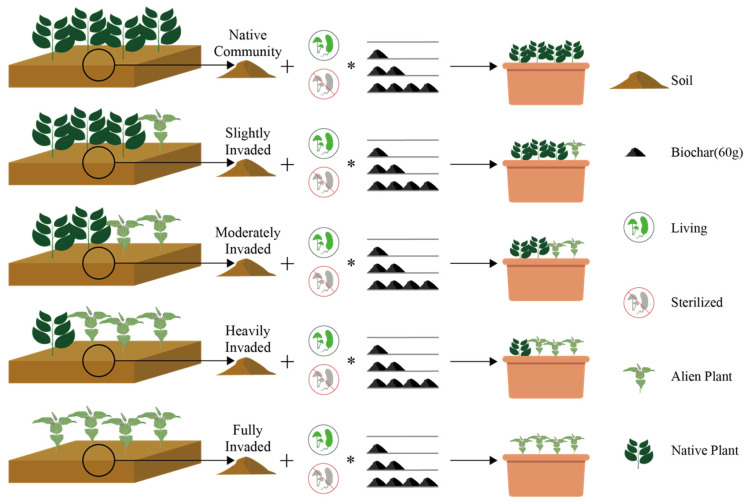
Design of the experiments: soil was collected from the original plots, treated with or without fungicide, and added with different levels of biochar, and the same plant combinations from the original plots were then transplanted into pots for the experiment. To distinguish from “sterilized”, we used the term “living” instead of “unsterilized” in the figures. The asterisk (*) denotes the interaction effect between sterilization and the addition of biochar.

To date, considerable research has been conducted on the use of biochar for remediating organic and heavy metal pollution [35]; however, research on the use of biochar to remediate soil after invasion has rarely been reported. The amount of biochar needed for soil remediation, whether different amounts are required for soils with varying levels of invasion, and the compatibility of biochar and fungicides are still largely unknown. Therefore, we used our focal alien invasive plant, *Chromolaena odorata*, along with three sympatric native plants, *Urena lobata*, *Abelmoschus manihot*, and *Achyranthes aspera*, to conduct the following experiments: (a) producing biochar from the invasive plant *C. odorata*; (b) excavating soil from plots with different levels of invasion, with or without the application of fungicide, with the addition of biochar (0, 60, 120, or 240 g), and using the original plant combinations from the plots for a pot experiment; (c) analyzing the remediation effects of fungicide and biochar on soils with different levels of invasion (Figure 1).

## 2. Results

As the degree of invasion increases, the nutrient elements phosphorus and potassium in the soil exhibit a decreasing trend. Simultaneously, the heavy metals manganese and lead also significantly decrease (Table 1). Invasive plants deplete these soil nutrients, thereby affecting the growth of subsequent plants. Even after removing invasive plants and restoring the native plant community, it is crucial to manage the legacy effects in the soil.

As illustrated in Table 2, the term ‘origin factor’ indicates whether the plant is invasive or native. The ‘sterilization factor’ represents the application of fungicide. ‘Invasive intensity’ describes the degree of invasion within the original community, while the ‘biochar factor’ pertains to the different levels of biochar application. The *p*-values for the interactions among origin × sterilization, origin × invasion intensity level, origin × biochar, sterilization × biochar, and origin × sterilization × invasion intensity level were all less than 0.05, indicating significant interactions between these factors. These interactions suggest that soil origin, fungicide application, invasion intensity, and biochar addition interactively influence the invasion of *C. odorata* (Table 2).

**Figure 2:**
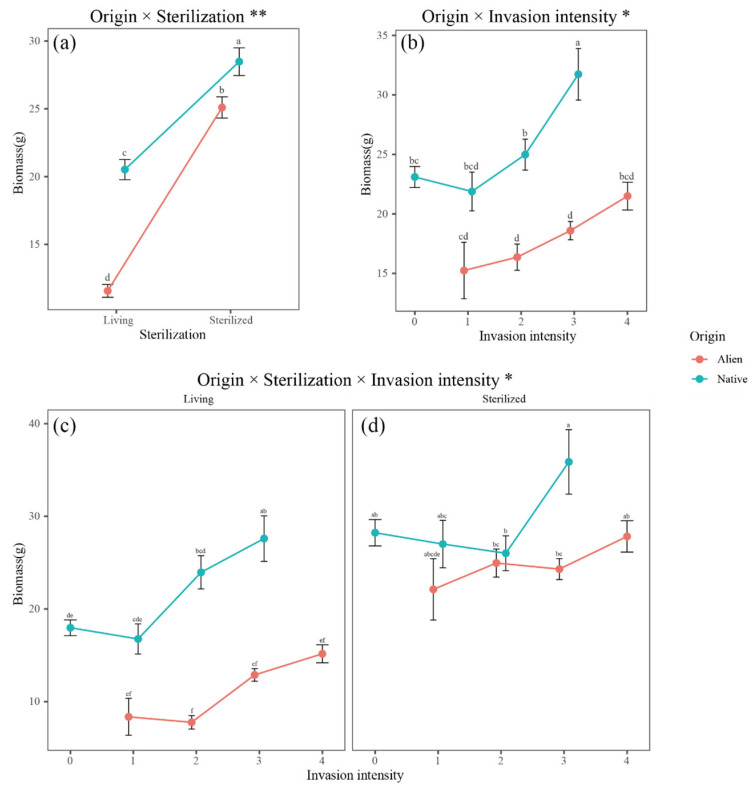
The interactive effects of (**a**) origin and sterilization; (**b**) origin and invasion intensity; and origin and invasion intensity on plant biomass in live soil (**c**) and sterilized soil (**d**). Points represent the mean biomass; error bars represent the means ± SEs. Letters indicate significant differences (*p* < 0.05). ** indicates 0.001 < *p* < 0.01, and * indicates 0.01 < *p* < 0.05.

Soil sterilization significantly increased the total biomass of both invasive and native plants (*p* < 0.001), with a steeper increase in biomass for alien plants than for native plants (Figure 2a). Native plant biomass was significantly greater under heavy invasion than under the other three invasion intensities. Under slight invasion, there was no significant difference in biomass between native and alien invasive plants. However, under moderate to high invasion, the biomass of native plants was significantly greater than that of invasive plants (Figure 2b). In unsterilized soil, the biomass of both native and alien invasive plants increased with the increasing invasion intensity, with that of native plants being significantly greater than that of invasive plants (Figure 2c). In sterilized soil, a significant difference in biomass between native and invasive plants was observed only under heavy invasion (Figure 2d).

**Figure 3:**
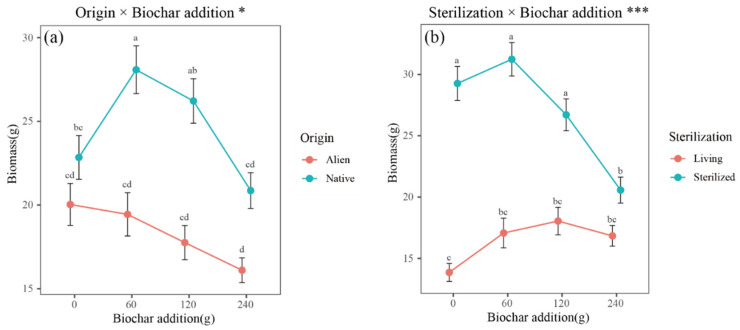
The effects of the interaction of (**a**) origin and biochar addition and (**b**) sterilization and biochar addition on plant biomass. Points represent the mean biomass. The error bars represent the means ± SEs. Letters indicate significant differences (*p* < 0.05). *** indicates *p* < 0.001, and * indicates *p* < 0.05.

With increasing biochar addition, the biomass of invasive plants gradually decreased, albeit not significantly, whereas the native plant biomass initially increased and then decreased. Significant differences in biomass between native and invasive plants were noted at biochar additions of 60 and 120 g (Figure 3a). The plant biomass generally initially increased but then decreased with the increasing biochar addition; however, this trend was not statistically significant in unsterilized soil. In sterilized soil, plant biomass significantly decreased with the addition of 240 g of biochar, which aligns closely with the change in biomass of plants grown in unsterilized soil with the same amount of biochar addition (Figure 3b).

**Figure 4:**
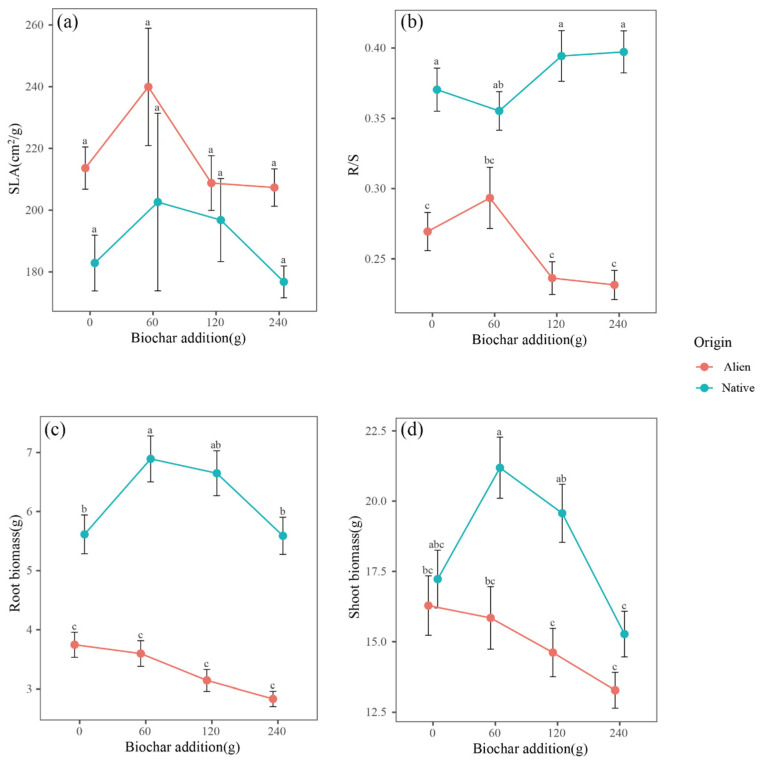
The effect of biochar addition on (**a**) specific leaf area, (**b**) root-to-shoot ratio, (**c**) belowground biomass, and (**d**) aboveground biomass. Points represent the mean of each indicator. The error bars represent the means ± SEs. Letters indicate significant differences (*p* < 0.05). SLA represents the specific leaf area, and R/S represents the ratio of root biomass to shoot biomass.

**Figure 5:**
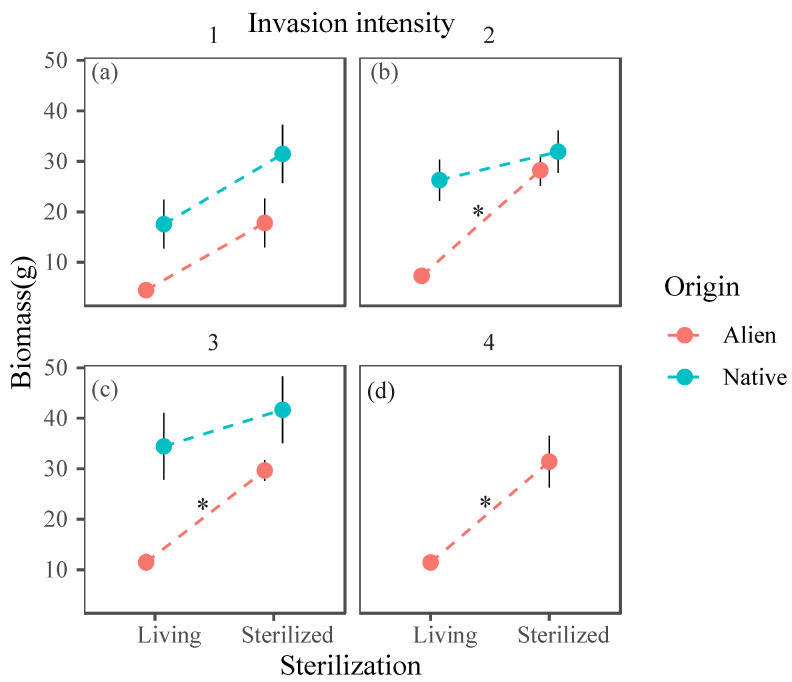
The effect of sterilization on the biomass of plants from different origins under different invasion levels at 60 g of biochar addition. Points represent the mean SLA (specific leaf area). Levels 1, 2, 3, and 4 represent the four invasion intensities: (**a**) slight, (**b**) moderate, (**c**) heavy, and (**d**) full. The error bars represent the means ± SEs. * Indicates *p* < 0.05.

To further explore the differences between native and invasive plants, we analyzed the specific leaf area (SLA), root biomass, shoot biomass, and root-to-shoot biomass ratio (R/S) of both plant types. Across all biochar addition levels, no significant differences were detected in the SLA of either alien invasive or native plants (Figure 4a). Compared to the control without biochar, the addition of 60 g of biochar significantly increased the root and shoot biomass of native plants (Figure 4c,d). Furthermore, with the addition of biochar at both 60 and 120 g, significant differences in shoot biomass were observed between native and invasive plants (Figure 4d). Under slight invasion, the combined application of 60 g of biochar and fungicide increased the biomass of both native and alien invasive plants, although not to a statistically significant degree (Figure 5a). Under moderate to heavy invasion, this combination significantly increased the biomass of alien invasive plants but only slightly increased that of the native plants (Figure 5b–d).

## 3. Discussion

### 3.1. Soil Legacy Effects of Invasive Plant Removal

The interaction between source and sterilization suggests that invasive plants and native plants had different influences on soil microbes within their respective experimental plots. Consequently, sterilization treatment led to varying responses in plants from different origins. Sterilization treatment significantly increased the total biomass of both invasive and native plants, which may indicate that, in the experimental plots, both plant types were subjected to negative plant–soil feedback. After sterilization, plants could re-recruit beneficial microbial communities, alleviating the negative impacts of existing soil microorganisms. The higher biomass increase rate observed in invasive plants than in native plants suggests that invasive plants may have experienced stronger negative plant–soil feedback due to the accumulation of soil pathogens within the invaded community (Figure 2a).

The accumulation of local pathogens suggests that invasive plants can suppress the growth of native plants by recruiting a large number of pathogens without having a negative impact on themselves [10]. However, research has shown that invasive plants often receive positive plant–soil feedback when invading new environments, but as the duration of plant invasion increases, the strength of positive feedback gradually decreases, leading to neutral or even negative feedback [36]. As the intensity of the invasion increases, the community may shift towards a monodominant state dominated by invasive plants. This shift can reduce biodiversity and potentially favor specialist pathogens that target specific species [37,38]. As the invasion intensity of *C. odorata* increases, the plants can regulate the composition and diversity of soil microorganisms through their roots. This regulation affects soil nutrients and the local plant community, thereby promoting its own growth and partially overcoming negative density constraints [39].

### 3.2. Effectiveness of Biochar in Remediation

Biochar modifies the physical structure and nutrient levels in the soil, thereby influencing the microbial recruitment by plants [40]. Our study revealed that biochar addition could significantly increase the biomass of native plants while having little effect on invasive plants (Figure 3a). The combined application of biochar with fungicide significantly improved plant growth in invaded communities (Figure 3b). However, the addition of 240 g of biochar eliminated the relative advantage of native plants over alien invasive plants. The reduction in plant biomass resulting from the addition of 240 g of biochar suggests a non-species-specific negative effect in which both alien invasive and native plants respond similarly to excessive biochar addition (Figure 3a). We speculate that applying more biochar than required may block soil pores, hindering root respiration and thereby inhibiting plant growth.

Since there was no significant interaction between the invasion intensity level and biochar or among origin, invasion intensity level, and biochar (Table 2), the appropriate amount of biochar can be determined for different invasion intensity levels of plant communities. We found that the addition of 60 g, equivalent to 2% of the soil mass, yielded the best soil remediation effect (Figure 3a). Compared to the unamended plants, the native plants presented significant growth improvements, whereas the invasive plants presented slight growth suppression. Treatment with 60 g of biochar appeared to particularly benefit native plants.

To explore the source of this preference, we further analyzed the specific leaf area (SLA), root biomass, shoot biomass, and root-to-shoot ratio (R/S). A lower root-to-shoot ratio indicates that plants allocate more biomass to their aboveground parts, and shoot competition has been shown to reduce community diversity [41]. A higher SLA allows for a shorter return on leaf investments and is strongly correlated with a high relative growth rate (RGR) [42]. It is believed that invasive plants prioritize growth over defense at invasion sites, resulting in a bias towards biomass allocation and investment strategies that facilitate rapid growth [43,44]. We found that, with the addition of 60 g of biochar, native plants allocated more resources aboveground and fewer resources belowground. This could be attributed to the biochar addition compensating for nutrient depletion resulting from the invasion of invasive plants and increasing soil nutrient conditions, thus allowing native plants to allocate more photosynthetic products to their photosynthetic parts.

Since origin × invasion intensity level × sterilization has a significant interaction effect, it is necessary to consider whether sterilization should be combined. In communities under slight invasion, sterilization could increase the biomass of native plants, alleviating soil nutrient depletion and the accumulation of soil-borne pathogens caused by alien invasive plant invasion. However, under relatively high invasion intensities, sterilization may not be necessary, as it may have significantly reduced the negative plant–soil feedback experienced by invasive plants while having a minimal effect on native plants, thus reducing the competitive advantage of native plants after biochar addition (Figure 5).

Further research is needed to understand why biochar addition facilitates the growth of native plants more effectively. However, in terms of application, incorporating biochar at 2% soil mass is recommended for mitigating the soil legacy effects of invasive plants. When invasive plants are removed from a slightly invaded community, converting them into biochar and adding them back into the soil, in combination with fungicide application, can help mitigate negative soil legacy effects on native plants and promote the recovery of native plant communities. After invasive plants are removed from moderately, heavily, and fully invaded communities, their conversion into biochar and addition to the soil can improve soil fertility and properties without the need for sterilization. The utilization of soil-borne pathogens accumulated by invasive plants to suppress their growth upon reinvasion can increase the relative competitive advantage of native plants over invasive plants.

Importantly, although this study is based on long-term field experiments, conclusions regarding the optimal amount of added biochar and the necessity of sterilization are derived from pot experiments conducted in a greenhouse. To apply both biochar and fungicide in practice, a series of field experiments is necessary to determine whether the required amounts of biochar and fungicide under field conditions align with the recommended dosages from these experiments.

This study provides a novel approach to addressing soil legacy effects caused by invasive plant *C. odorata* through the use of biochar derived from its biomass. To our knowledge, this is the first study to propose utilizing biochar made from invasive plants as a means of addressing both the safe disposal of biomass after their removal and the subsequent restoration of soils they have degraded. Compared to traditional methods such as open burning, anaerobic digestion used in this study offers a cleaner and more sustainable alternative. Operating under oxygen-limited conditions, this method reduces carbon oxidation, significantly reducing CO₂ emissions and harmful gases such as carbon monoxide. This ensures a more efficient transformation of biomass into stable carbon forms, enhancing the environmental and economic feasibility of managing invasive species.

In addition, this study explores the integration of multiple factors—such as invasion intensity, soil sterilization, and biochar application levels—into soil restoration strategies. By systematically examining the interactions among these factors, our findings suggest that restoration strategies could benefit from being adjusted to the specific invasion intensity of the invaded community. This multifactorial approach offers insights into the adaptive management of invaded ecosystems, contributing to more effective restoration under diverse ecological conditions.

These findings lay a foundation for the development of environmentally friendly and dual-purpose solutions to combat invasive species, demonstrating the potential for transforming ecological threats into valuable resources while simultaneously promoting the recovery of the native community.

## 4. Materials and Methods

### 4.1. Invasive Plant Community Plots

This research is based on a platform specifically constructed for the long-term monitoring of dynamics within invaded plant communities. Located at the Xishuangbanna Tropical Botanical Garden, Chinese Academy of Sciences (21°56′ N, 101°25′ E, 560 m a.s.l.) in Yunnan Province, Southwest China, this platform experiences a mean annual temperature of 21.7 °C, with July being the hottest month at 25.3 °C and January being the coolest month at 15.6 °C. The annual precipitation averages 1557 mm, with a dry period spanning from November to April [45]. The platform consists of 2 × 2 m plots, each containing four individuals of the plants. The species combinations and their corresponding invasion intensity levels for each plot were as follows: (1) four *C. odorata* plants, defined as ‘Invasion intensity level 4, fully invaded’; (2) three *C. odorata* plants and one native plant (*Ab. manihot*, *Ac. Aspera*, or *U. lobata*), defined as ‘Invasion intensity level 3, heavily invaded’; (3) two *C. odorata* plants and two native plants (one *Ab. manihot* and one *U. lobata*, one *Ab. manihot* and one *Ac. aspera*, or one *Ac. aspera* and one *U. lobata*), defined as ‘Invasion intensity level 2, moderately invaded’; (4) one *C. odorata* plant and one native plant of each species, defined as ‘Invasion intensity level 1, slightly invaded’; (5) four plants of each native species (four *Ab. manihot*, four *Ac. aspera*, and four *U. lobata*), defined as ‘Invasion intensity level 0, native community’ (Figure 1). Each combination was replicated three times, for a total of 33 plots.

### 4.2. Plant Material

The alien invasive plant *C. odorata* and three native plants, *U. lobata*, *Ab. manihot*, and *Ac. Aspera*, were used for the experiment. *C. odorata* is one of the most noxious alien invasive plants in Southern China [46] and is commonly found in the Xishuangbanna region where our experiment was conducted.

In April 2023, seeds of *C. odorata*, *Ab. manihot*, *Ac. aspera*, and *U. lobata* were collected from invaded community plots on the monitoring platform and sown into nursery trays measuring 50 × 50 × 10 cm using a mixture of organic substrate (Pindstrup, Denmark) and river sand at a ratio of 2:1. The seedlings were watered daily. After one month of nurturing, when all the seedlings reached approximately 10 cm in height, they were ready for transplantation into pots.

### 4.3. Biochar Preparation

We collected the biomass of *C. odorata* in the invaded areas, sun-dried it, and then processed it into biochar. Biochar production was carried out using the anaerobic digestion method in a carbonization furnace. The temperature inside the furnace was gradually increased from room temperature at a rate of 5 °C per minute until reaching 400 °C; after which, it was maintained for 2 h. The biochar was physically modified manually by placing it in a plastic bag, using a hard object to pestle the biochar, breaking it into smaller particles, and then sieving it through a 60-mesh screen to be used for subsequent pot experiments.

### 4.4. Pot Experiment

Soil samples were collected from the surface layer (0–10 cm) of each plot, sieved through a 5-mm mesh, and then filled into square pots measuring 30 × 30 × 20 cm. To investigate the role of soil microbiota in the competitive interactions among plants, half of the pots underwent sterilization treatment using the fungicide dazomet (Aladdin, Shanghai, China). This treatment was intended to disrupt or eliminate the soil microbial communities of the original plots, thereby isolating the effects on the subsequent plant growth. The other half of the pots were left unsterilized as controls to preserve the original soil microbial conditions.

To explore the impact of biochar addition on plant growth and competitive outcomes, biochar derived from *Chromolaena odorata* biomass was applied at four levels: 0, 60, 120, and 240 g per pot, corresponding to 0%, 2%, 4%, and 8% of the soil mass, respectively.

A total of 1056 previously cultivated seedlings (480 *C. odorata* plants and 192 plants of each native species) were transplanted into the pots according to the original plot species combination, totaling 264 pots (33 species combinations × 2 sterilization conditions × 4 biochar addition levels). Watering, weeding, and insecticide spraying were performed as necessitated by the growth conditions of the potted plants to ensure normal growth.

### 4.5. Data Collection

In September 2023, after 4 m of growth, the biomass of each potted plant was harvested. The leaves, stems, and roots of each plant were harvested separately; dried in a drying oven (DHG-9620A, Yiheng, Shanghai, China) at 70 °C for 72 h; and weighed. The leaf area was determined using a Li-3000A leaf area meter (LI-COR, Lincoln, NE, USA).

The shoot biomass (aboveground biomass) was calculated as the sum of the leaf biomass and stem biomass. The specific leaf area (SLA) was calculated as the leaf area divided by the leaf biomass. The root-to-shoot ratio (R/S) was calculated as the root biomass divided by the shoot biomass.

The elemental content in the soil was determined using wavelength dispersive X-ray fluorescence (WDXRF) spectrometry [47].

### 4.6. Data Analysis and Plotting

All analyses and plotting were conducted in R (version 4.3.3) [48]. Plant biomass, measured as the total dry weight (including leaves, stems, and roots), was used as the primary response variable to assess the competitive outcomes over the experiment’s duration.

The analysis aimed to determine whether biochar addition, fungicide treatment, or their interactions with the invasion intensity levels had differential effects on the growth of native and invasive plants. A mixed-effects model was constructed using the lmerTest package [49], where the fixed factors included plant origin (native vs. invasive), invasion intensity level, fungicide treatment, and biochar addition level. Species was treated as a random factor to account for the inherent growth differences among species.

To explore the interactions between fixed factors, analysis of variance (ANOVA) was performed on this model to identify complex relationships, such as how biochar might interact with fungicide to influence plant growth. To compare differences between groups, a separate ANOVA was conducted on the biomass data, followed by multiple comparisons using the Tukey’s HSD method (confidence level = 0.95). Plots were drawn using the ggplot2 package [50].

## 5. Conclusions

In this study, we produced biochar from *Chromolaena odorata* and incorporated it, in combination with fungicide, into the soil of plant communities experiencing varying levels of invasion intensity. Our findings indicate that the addition of biochar at 2% soil mass significantly increased the biomass of native plants, mitigating the soil legacy effects within invaded communities at different invasion intensities. Moreover, it slightly reduced the biomass of alien invasive plants, thereby giving native plants a competitive advantage. When the invasion intensity is low, the application of fungicide in conjunction with biochar can enhance the recovery of native plant communities. Further extensive studies are necessary to evaluate the use of biochar and fungicide in field settings, as well as to assess the safety and ecological impacts of this approach.

## Figures and Tables

**Table 1:** The mean ± standard error (SE) of the soil element concentrations is presented. Letters below the values indicate statistical significance at *p* < 0.05. The numbers 0, 1, 2, 3, and 4 in the table header denote the levels of invasion: native community, slightly invaded, moderately invaded, heavily invaded, and fully invaded.

Elements	Invasion Intensity				
	0	1	2	3	4
P (mg/kg)	869.39 ± 93.08	990.87 ± 376.58	717.77 ± 115.45	680.18 ± 127.42	496.4 ± 53.84
	a	a	a	a	a
S (mg/kg)	158.17 ± 3.35	198.3 ± 15.01	185.62 ± 9.72	199 ± 15.91	237.87 ± 15.14
	b	ab	ab	ab	a
Mn (mg/kg)	638.04 ± 28.15	266.33 ± 74.03	595.76 ± 82.84	425.21 ± 122.44	113.8 ± 11.89
	a	ab	a	ab	b
Pb (mg/kg)	25.14 ± 3.02	12.73 ± 4.68	15.97 ± 1.59	15.02 ± 2.7	9.97 ± 2.21
	a	ab	ab	ab	b
Ca (g/kg)	2.27 ± 0.09	0.88 ± 0.37	1.63 ± 0.28	1.5 ± 0.43	0.47 ± 0.02
	a	ab	ab	ab	b
K (g/kg)	13.57 ± 0.1	13.07 ± 1.95	13.24 ± 0.46	12.07 ± 0.64	10.69 ± 0.78
	a	a	a	a	a
Al (g/kg)	68.24 ± 0.58	91.26 ± 6.93	84.91 ± 3.56	78.08 ± 4.48	84.87 ± 8.94
	b	a	a	ab	ab

**Table 2:** ANOVA table for the mixed-effects model, where Factor = names of fixed factors, and NumDF = numerator degrees of freedom. *p* < 0.05 indicates that this factor has a significant effect on plant biomass. Interactions between fixed factors are denoted by a ×, and significant effects (*p* < 0.05) are highlighted in bold.

Factor	NumDF	*p*
Origin	1	0.295
**Invasion intensity** ***	4	<0.001
**Sterilization** ***	1	<0.001
**Biochar** **	3	0.002
**Origin × Invasion intensity** *	2	0.049
**Origin × Sterilization** **	1	0.002
Invasion intensity × Sterilization	4	0.460
**Origin × Biochar** *	3	0.032
Invasion intensity × Biochar	12	0.515
**Sterilization × Biochar** ***	3	<0.001
**Origin × Invasion intensity × Sterilization** *	2	0.012
Origin × Invasion intensity × Biochar	6	0.489
Origin × Sterilization × Biochar	3	0.926
Invasion intensity × Sterilization × Biochar	12	0.986
Origin × Invasion intensity × Sterilization × Biochar	6	0.920

*** indicates *p* < 0.001, ** indicates 0.001 < *p* < 0.01, and * indicates 0.01 < *p* < 0.05.

## Data Availability

The data presented in this study are available in this article.
